# Genome-wide identification and characteristic analysis of the downstream melatonin metabolism gene *GhM2H* in *Gossypium hirsutum* L.

**DOI:** 10.1186/s40659-021-00358-y

**Published:** 2021-11-04

**Authors:** Yuexin Zhang, Jing Wang, Xiugui Chen, Xuke Lu, Delong Wang, Junjuan Wang, Shuai Wang, Chao Chen, Lixue Guo, Waqar Afzal Malik, Yapeng Fan, Cun Rui, Ruifeng Cui, Qinqin Wang, Yuqian Lei, Wuwei Ye

**Affiliations:** State Key Laboratory of Cotton Biology/Institute of Cotton Research of Chinese Academy of Agricultural Sciences, Zhengzhou Research Base, School of Agricultural Sciences, Zhengzhou University Research Base, Zhengzhou University/Key Laboratory for Cotton Genetic Improvement, MOA, Anyang, Henan China

**Keywords:** M2H, 2-hydroxymelatonin, Melatonin, Gene family, Abiotic stress, Cotton

## Abstract

**Background:**

Melatonin 2-hydroxylase (M2H) is the first enzyme in the catabolism pathway of melatonin, which catalyzes the production of 2-hydroxymelatonin (2-OHM) from melatonin. The content of 2-hydroxymelatonin in plants is much higher than that of melatonin. So M2H may be a key enzyme in the metabolic pathway of melatonin.

**Method:**

We conducted a systematic analysis of the *M2H* gene family in *Gossypium hirsutum* based on the whole genome sequence by integrating the structural characteristics, phylogenetic relationships, expression profile, and biological stress of the members of the *Gossypium hirsutum M2H* gene family.

**Result:**

We identified 265 *M2H* genes in the whole genome of *Gossypium hirsutum*, which were divided into 7 clades (clades I-VII) according to phylogenetic analysis. Most *M2H* members in each group had similar motif composition and gene structure characteristics. More than half of *GhM2H* members contain ABA-responsive elements and MeJA-responsive elements. Under different stress conditions, the expression levels of the gene changed, indicating that *GhM2H* members were involved in the regulation of abiotic stress. Some genes in the *GhM2H* family were involved in regulating melatonin levels in cotton under salt stress, and some genes were regulated by exogenous melatonin.

**Conclusion:**

This study is helpful to explore the function of GhM2H, the downstream metabolism gene of melatonin in cotton, and lay the foundation for better exploring the molecular mechanism of melatonin improving cotton's response to abiotic stress.

**Supplementary Information:**

The online version contains supplementary material available at 10.1186/s40659-021-00358-y.

## Introduction

In 1995, two research organizations detected melatonin in vascular plants [1, 2], opening the door to the study of melatonin in plants. Subsequent studies have shown that melatonin is widely present in plants, and the term "phytomelatonin" was proposed in 2004 [3]. In recent years, there have been more and more researches on plant melatonin, and scientific researchers have been studying it more and more deeply. The concept of plant melatonin as an important plant hormone has gradually been accepted [4]. Several studies have shown: melatonin can be used as plant growth regulators, promote the growth, root elongation [5], seed germination [6], photoperiod [7] and photosynthesis [8]; as an effective antioxidant, scavenging free radicals and up-regulating various antioxidant enzymes [9]; as an anti-stress hormone, it gives plants resistance to drought, ultraviolet radiation, heavy metals, salt ions and other abiotic stress and some biological stress [10]. This shows that the study of plant melatonin is of great significance.

In plants, the synthetic substrate of melatonin is tryptophan [11], and melatonin is produced through four enzymatic reactions [12]. The four enzymes are TDC (tryptophan decarboxylase) [13], T5H (tryptamine 5-hydroxylase) [14], SNAT (serotonin *N*acetyltransferase) [15], ASMT (*N-*acetylserotonin methyltransferase) [16]. Furthermore, the registered COMT (Caffeic acid o-methyltransferase) leaves of *Arabidopsis* have the activity of ASMT enzyme [17]. Because the conservation of plant melatonin biosynthesis genes contradicts the low concentration of melatonin in plants, it may be rapidly catabolized into other substances. But the metabolic pathway of melatonin has not been studied clearly. And the reported genes related to melatonin catabolism include *M2H* (melatonin 2-hydroxylase) [18], IDO (indoleamine 2,3-dioxygenase) [19], M3H (melatonin 3-hydroxylase) [20].

Many metabolites of melatonin have been identified in animals, include N^1^-acetyl-N^2^-formyl-5-methoxykynuramine (AFMK), 2-Hydroxymelatonin (2-OHM),6-hydroxymelatonin (6-OHM), 4-hydroxymelatonin (4-OHM), and cyclic 3-hydroxymelatonin (3-OHM) [21, 22]. The metabolites converted into melatonin in plants include 2-OHM (2-Hydroxymelatonin) [23], 3-OHM (cyclic 3-hydroxymelatonin) [20], AFMK (N^1^-acetyl-N^2^-formyl-5-methoxykynuramine) [24]. Among them, 2-OHM is the main metabolite of melatonin, and its concentration in plants is much higher than that of melatonin, indicating that it has important physiological effects [25]. It has been demonstrated that in addition to melatonin, its precursors and the metabolite 2-OHM are also involved in plant stress resistance [26]. At this stage, most of the research has focused on the synthesis pathway of melatonin, and the catabolism pathway of melatonin is still unclear. Therefore, it is of great significance to study the catabolic pathway of melatonin for improving the stress resistance of plants.

In plants, hydroxylation of primary and secondary metabolites is performed by 2-ODD and monooxygenase (P450) dependent on cytochrome P450 [27]. In 2015, the melatonin 2-hydroxylase (*M2H*) gene, which catalyzes the conversion of melatonin to 2-hydroxylated melatonin, was cloned for the first time from 35 2-ODD family members in the *Oryza sativa* genome [18]. As the first key enzyme in the catabolism pathway of melatonin, M2H catalyzes the production of 2-hydroxymelatonin (2-OHM). Its activity was higher than that of melatonin synthase SNAT and ASMT [28, 29]. Therefore, the metabolic rate of melatonin is higher than the synthesis rate, and the melatonin content under normal conditions is kept at a very low level, thereby regulating plant growth and development. Melatonin 2-hydroxylase (*M2H*) is an important factor in the maintenance of endogenous melatonin content in plants. *M2H* RNAi *Oryza sativa* produced more melatonin after cadmium treatment to tolerate cadmium stress, while showing resistance to salt stress [30].

In this study, the *M2H* gene family in *Gossypium hirsutum* was systematically analyzed based on the whole genome sequence by integrating the structural characteristics, phylogenetic relationships, exon/intron structures, expression profiles and evolutionary relationships of members of the *M2H* gene family. In this study, 265 *M2H* genes were identified in the whole genome of *Gossypium hirsutum*. We analyzed the expression pattern of *M2H* genes under cold, heat, salt and PEG stress. The results of this study provide a reference for further analysis of the function of *GhM2H* gene and exploration of melatonin metabolism.

## Results

### Identification of *GhM2H *Gene Family Members in *Gossypium hirsutum*

The protein sequences and nucleic acid sequences of four genes (AK067086, AK065790, AK119413, AK101447) that have been reported to have 2-melatonin hydroxylase activity in rice were used as query sequences for comparison using the software local Blast. We identified the candidate genes of the *M2H* gene in the *Gossypium hirsutum* genome, deleted the incomplete genes in the conserved domains 2OG-FeII_Oxy, DIOX_N (PF03171.20, PF14226.6), and renamed these genes according to their positions on the chromosomes *GhM2H*1-*GhM2H*265, and then we analyzed and predicted the physical properties of these genes, including ID, isoelectric point, molecular weight, protein length and subcellular location (Additional file 1).

265 *M2H* genes were identified in the *Gossypium hirsutum* genome. The protein sequences encoded by these genes range from 212 (*GhM2H*199) to 418 (*GhM2H*38) amino acids, with isoelectric points ranging from 4.67 (*GhM2H*200) to 9.42 (*GhM2H*81). The MW ranges from 25.14 (*GhM2H*208) kDa to 47.33 (*GhM2H*37) kDa. The subcellular location predicts that 217 genes are in the cytoplasm, 1 gene is outside the cell, 44 genes are in the outer membrane, and 29 genes are in the intermembrane.

### Phylogenetic analysis of *GhM2H*

**Fig. 1:**
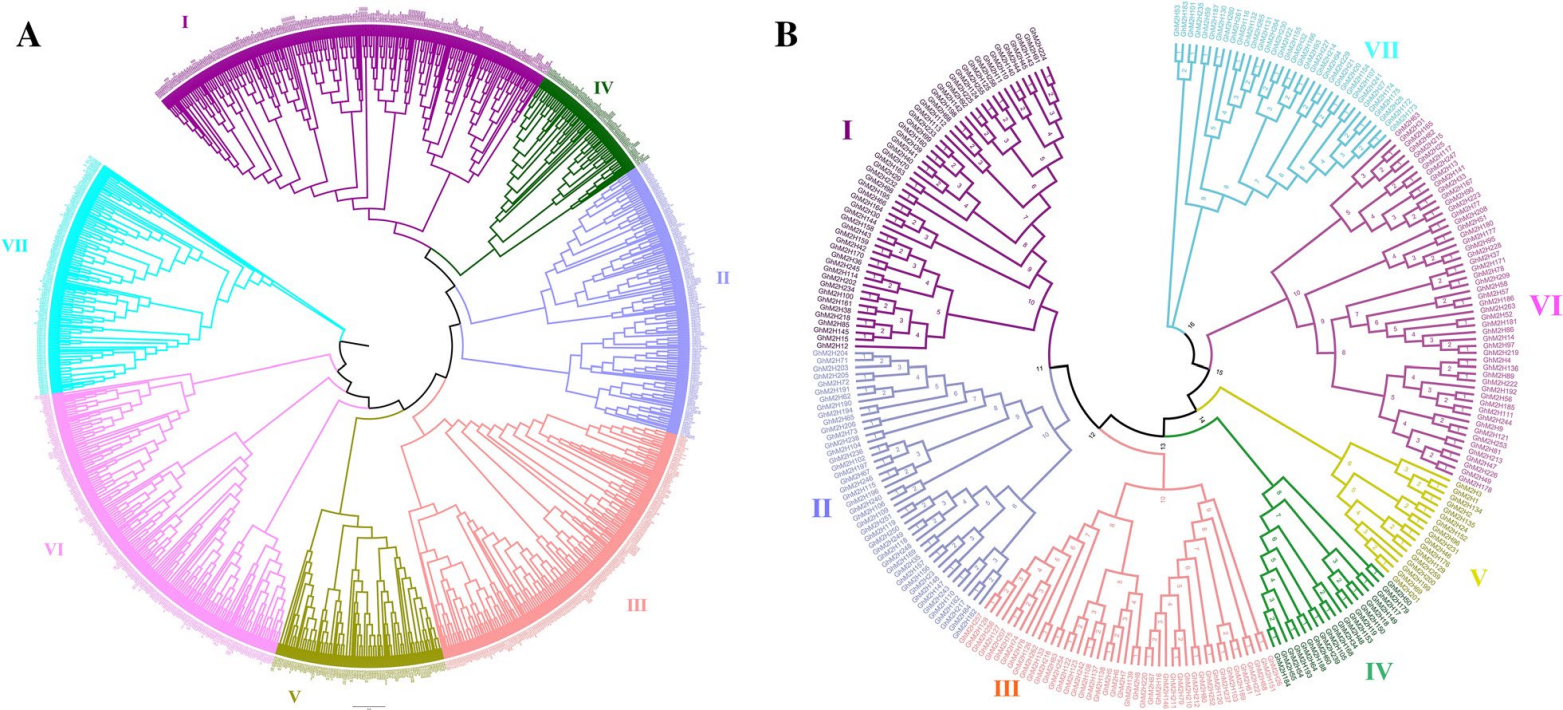
Two rooted phylogeny trees constructed using MEGA-7 by the Neighbor-Joining (NJ) method. Bootstrap values (above 50%) from 1000 replicates are indicated at each node. **A** Phylogenetic relationship of 1185 *M2H* genes identified from 4 plants. **B** Phylogenetic relationship of the 880 identified *M2H* genes from four *Gossypium hirsutum*

To investigate the evolutionary relationship of plant M2Hs, we compared the amino acid sequences of cotton M2Hs with those of *Arabidopsis*, *Oryza sativa* and *Theobroma cacao*. And a total of 1185 protein sequences (265 from *Gossypium hirsutum*, 272 from *Gossypium barbadense*, 169 from *Gossypium arboretum*, 174 from *Gossypium raimondii*, 97 from *Arabidopsis*, 87 from *Oryza sativa*, 121 from *Theobroma cacao*) were used to construct a phylogenetic tree (Fig. 1A). The M2H proteins of these four species are distributed in almost every clade. The M2H gene phylogenetic tree of these plants is mainly divided into seven branches, which are randomly distributed. Among them, clade IV has the least members (79), clade I has the most members (253), and clades II, III, V, VI, VII contains 171, 208, 105, 222, and 147 genes respectively. Interestingly, in *Arabidopsis*, *Oryza sativa* and *Theobroma cacao*, the M2H proteins of the four cotton species have corresponding homologous genes in each clade, indicating that the M2H proteins of these plants are closely related to each other. Phylogenetic analysis shows that the amount of *M2H* in *Gossypium hirsutum* is more than twice that of *Theobroma cacao*, *Oryza sativa* and *Arabidopsis thaliana*, and it has undergone significant gene family amplification during evolution [31]. In phylogenetic trees, we found that gene pairs of *GhM2H* and Gb*M2H* were always clustered together, which could be used as evidence of gene duplication. Meanwhile, the *M2H* protein of tetraploid cotton (*Gossypium hirsutum* and *Gossypium barbadense*) and diploid cotton (*Gossypium arboreum* and *Gossypium raimondii*) were congealed, confirming that *Gossypium hirsutum* and *Gossypium barbadense* were the result of a cross between *Gossypium arboreum* and *Gossypium raimondii*.

In order to further study the evolutionary relationship of *M2H* in *Gossypium hirsutum*, we constructed an evolutionary tree of the intraspecific *M2H* gene of *Gossypium hirsutum* (Fig. 1B). The *M2H* gene in *Gossypium hirsutum* was found in all seven clades. *M2H* genes have been found in the seven clades in *Gossypium hirsutum*, clade I, VI highest proportion (there are 53 *GhM2H*), clade II contains 43 *GhM2H*, clade III contains 44 *GhM2H*, clade VII contains 35 *GhM2H*, clade IV contains 20 *GhM2H* and clade V only 12 *GhM2H*. These results indicate that clade I is an ancient *M2H* gene group with the largest number of *M2H* members in almost all plants. In the phylogenetic tree, two genes are gathered together to form a gene pair, forming a total of 107 gene pairs. In each gene pair, one gene is from the A sub-genomes and the other is from the D sub-genomes.

### Chromosomal location of *GhM2H* members

**Fig. 2:**
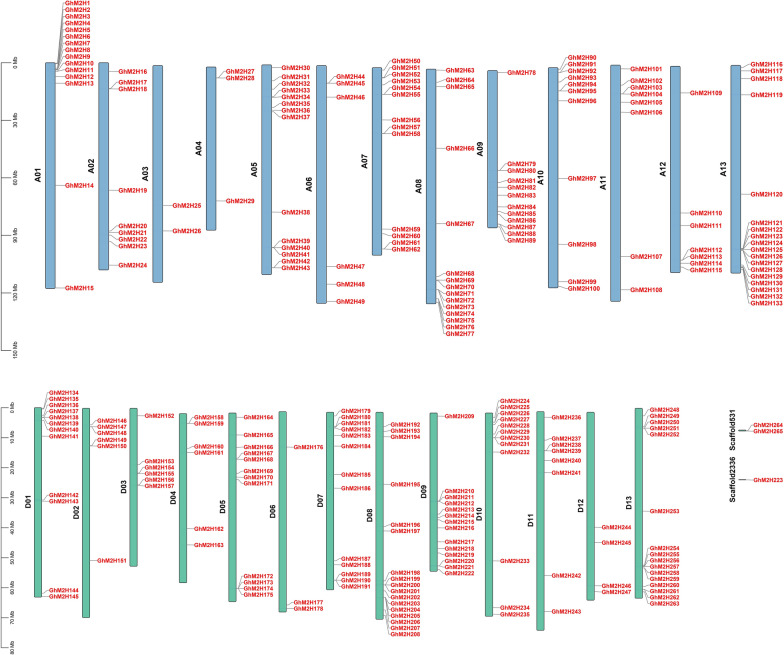
Chromosome location of *GhM2H*

To study the chromosomal distribution of members of the *GhM2H* gene family, we mapped the physical location of these genes on cotton chromosomes (Fig. 2). 262 genes are mapped to 26 chromosomes, which are unevenly distributed, and 3 genes (*GhM2H*223, *GhM2H*264, *GhM2H*265) are mapped to scaffold. The number of *M2H* genes on each chromosome is between 2 and 17. There are only 2 genes on chromosome A03, 3 genes on chromosomes A04 and D06; 18 genes on chromosome A13, 17 genes on chromosome D13, and 15 genes on chromosome A01 and A08. There is one gene (*GhM2H*223) on Scaffold2336, and two genes (*GhM2H*264, *GhM2H*265) on scaffold531.

### Analysis of Ka/Ks

In order to explore the impact of Darwin's selection on the differentiation of repetitive genes, an aligned sequence covering more than 80% of the longest gene and sequence similarity of more than 70% was used as the criterion for inferring gene repetition events. According to this gene duplication standard, we used the software TBtools to calculate the Ka/Ks ratio of these gene pairs (Additional file 2). In *Gossypium hirsutum*, we calculated the Ka/Ks value of 252 pairs of genes. It is generally believed that Ka/Ks = 1 is a neutral selection, Ka/Ks < 1 is a purifying selection, and Ka/Ks > 1 is a positive selection. The Ka/Ks values of these gene pairs are all less than 1. Among these genes, 233 pairs of genes have Ka/Ks values between 0–0.5, and 19 pairs of genes have Ka/Ks values between 0.5–99. We hypothesized that the *M2H* gene family of cotton underwent strong purification selection after fragment repetition and genome-wide duplication, with limited functional differences.

### Analysis gene duplication and collinearity

**Fig. 3:**
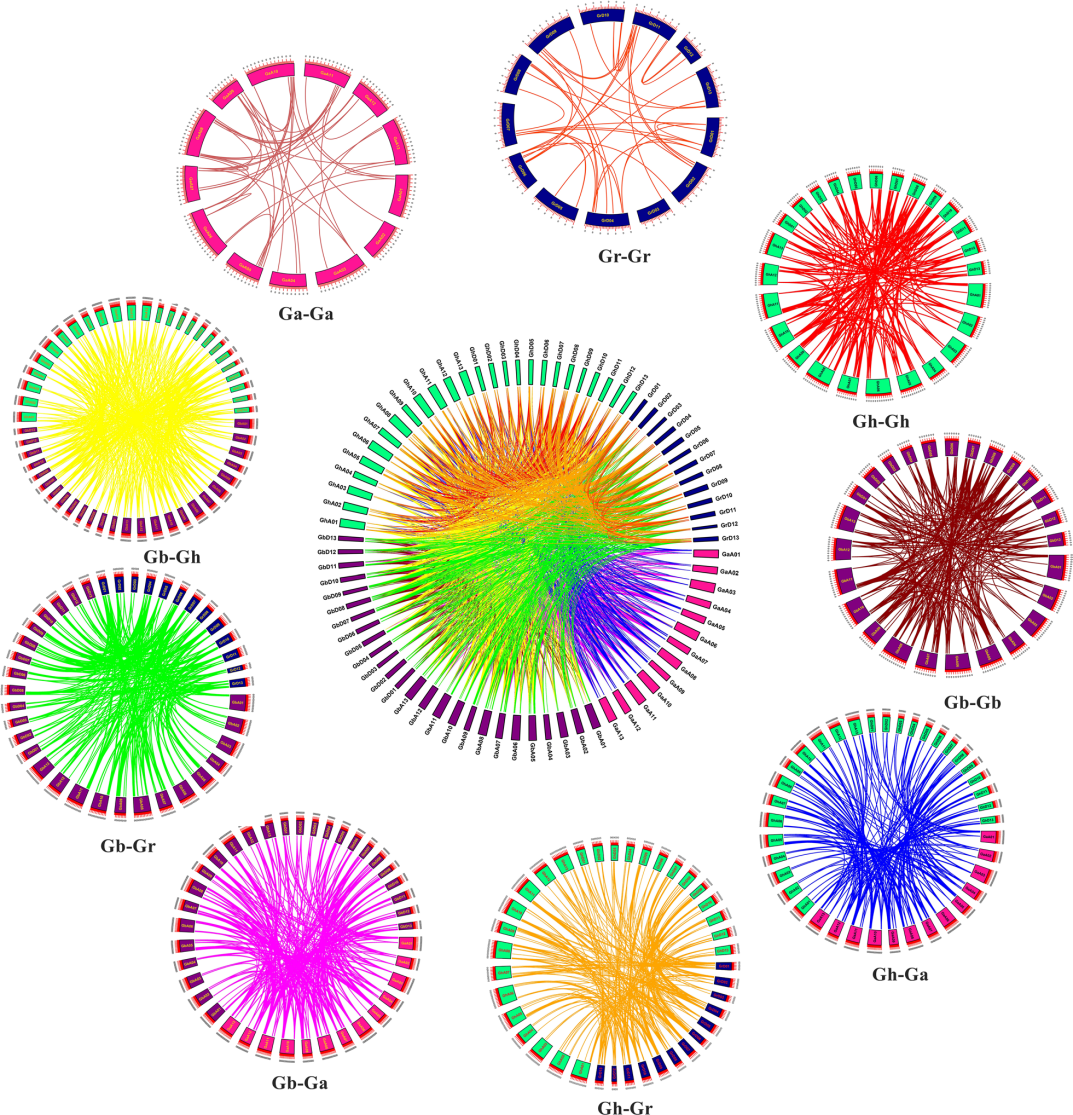
Syntenic relationship of duplicated genes pairs from four cotton species (*G. hirsutum, G. barbadense, G. arboreum and G. raimondii*). Chromosomal lines represented by various colors indicates the syntenic regions around the *M2H* genes

Replication events play an important role in gene amplification. Replication events include genome-wide replication, fragment replication and tandem replication. Through the homology analysis of the *M2H* genes of four cotton species (*Gossypium hirsutum*, *Gossypium barbadense*, *Gossypium arboreum*, *Gossypium raimondii*), the positional relationship of the homologous *M2H* genes of the four cotton species was visualized (Fig. 3). The adjacent genes on the same chromosome belong to tandem duplication, and the remaining genes from the same genome belong to fragment duplication [32]. The remaining genes from different genomes and sub-genomes belong to genome-wide replication. Gene duplication events are one of the main contributors to evolutionary dynamics, and they have a major impact on genome rearrangement and expansion. We identified homologous gene pairs in GhAt, GhDt, GbAt and GbDt sub-genomes of two tetraploid cotton and A and D genomes of two diploid cotton. Collinearity shows that the At and Dt sub-genomes of the two tetraploid cottons have several gene loci that are highly conserved.

The genes connected by the lines of the same color represent the same gene. In Fig. 3, we can see that many chromosomes in the GhAt/GhDt, GbAt/GbDt sub-genomes and the A and D genomes are connected by the same color line, namely GhAt/GhDt and GbAt/GbDt sub-genomes with *M2H* homologous genes in the A and D genomes, indicating that these genomes/sub-genomes are related in evolution, and most of the *M2H* genes are preserved in the evolution of polyploidy. We made a comparison between the genomes and sub-genomes of Ga-ga, Ga-Gb, Ga-Gr, Ga-Gh, Gb-Gb, Gb-Gr, Gb-Gh, Gr-Gr, Gr-Gh, and Gh-Gh, and identified a total of 3397 orthologous/paralogous gene pairs, 150 pairs of repeated genes have tandem duplication, 699 pairs of repeated genes have fragment duplication, and the remaining 2548 pairs of duplicate genes have genome-wide duplication.

### Analysis gene structure and motif

**Fig. 4:**
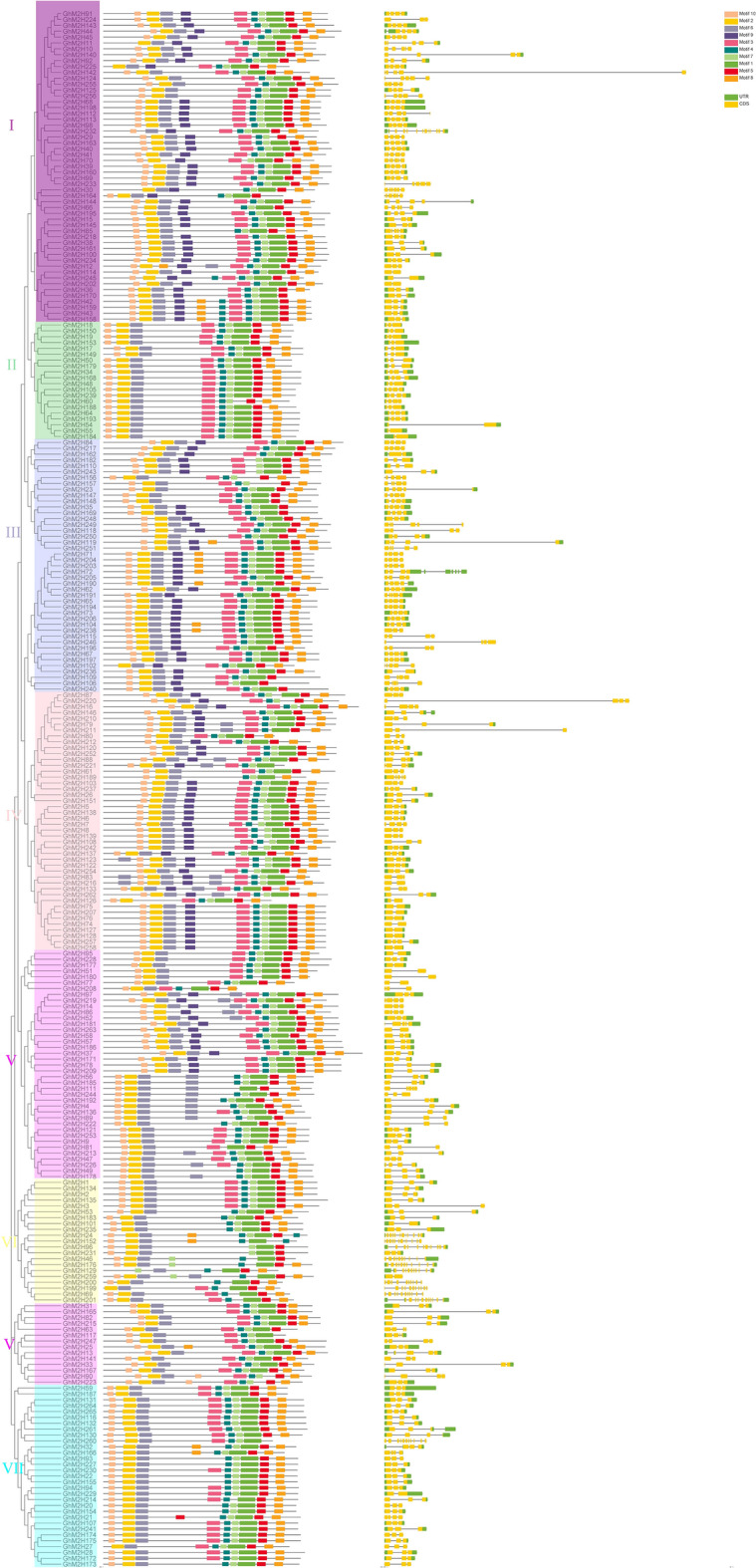
The evolutionary relationship of *GhM2H* in *Gossypium hirsutum*, the association analysis of motif composition and gene structure

To further understand the possible structural evolution of *GhM2H*, we constructed an association analysis of *GhM2H* members with phylogenetic tree, gene structure, and motif. We used the protein sequences of *GhM2H* members to construct a phylogenetic tree, obtained the gene structure of *GhM2H* members in the whole-genome annotation file of *Gossypium hirsutum*, submitted the protein sequences of *GhM2H* members to online tool MEME for conservative motif prediction, and used TBtools for association analysis (Fig. 4).

From the results of gene structure and phylogenetic tree, we can see that similar genes are clustered together in the same group of phylogenetic trees. The number of exons in each gene ranges from 2 to 12. In most cases, the two genes in a gene pair have similar exon–intron knot structure and length. There are also some genetic structures and pairs of genes that differ in length, such as: *GhM2H*119/*GhM2H*251, *GhM2H*108/*GhM2H*243, *GhM2H*220/*GhM2H*87, etc. There are 74 genes without introns, 29 genes have only one intron, and 4 genes have 12 exons. The number of gene exons in different clades is different, but most *GHM2H* members of the same clade have the same exon–intron structure. We found that the genetic structure of *GhM2H* gene has a strong relationship with phylogeny on the basis of evolution.

The online tool MEME was used to analyze the protein sequence of *GhM2H* members to obtain a total of 10 motifs, and proteins with the same motif composition were preferentially clustered together. As shown in Fig. 4, most of the *GhM2H* members of the same clade, especially the closely related members, often have similar motif composition. All protein motifs are distributed in the GhM2H protein, and the motif of each protein ranges from 6 to 10. In addition, the two genes in most gene pairs have the same motif composition, which means that they are functionally similar at the protein level. We found that most GhM2H proteins contain Motif10, 2, 6, 4, 7, 1, 5, 8. Almost all the GhM2H proteins of clades I, II, and III consist of 10 motifs, while some of the GhM2H proteins in clades IV, V, VII and VI have no motif 9. Interestingly, *GhM2H*129 only has motif 6, 4, 7, 1, 5, 8, and the motif composition of *GhM2H*259, which belongs to the same gene pair, has obvious differences, and there are also obvious differences in the structure of the two genes. We speculate that GhM2H129 may have lost some of its functions during evolution.

### Analysis of *GhM2H* promoter

**Fig. 5:**
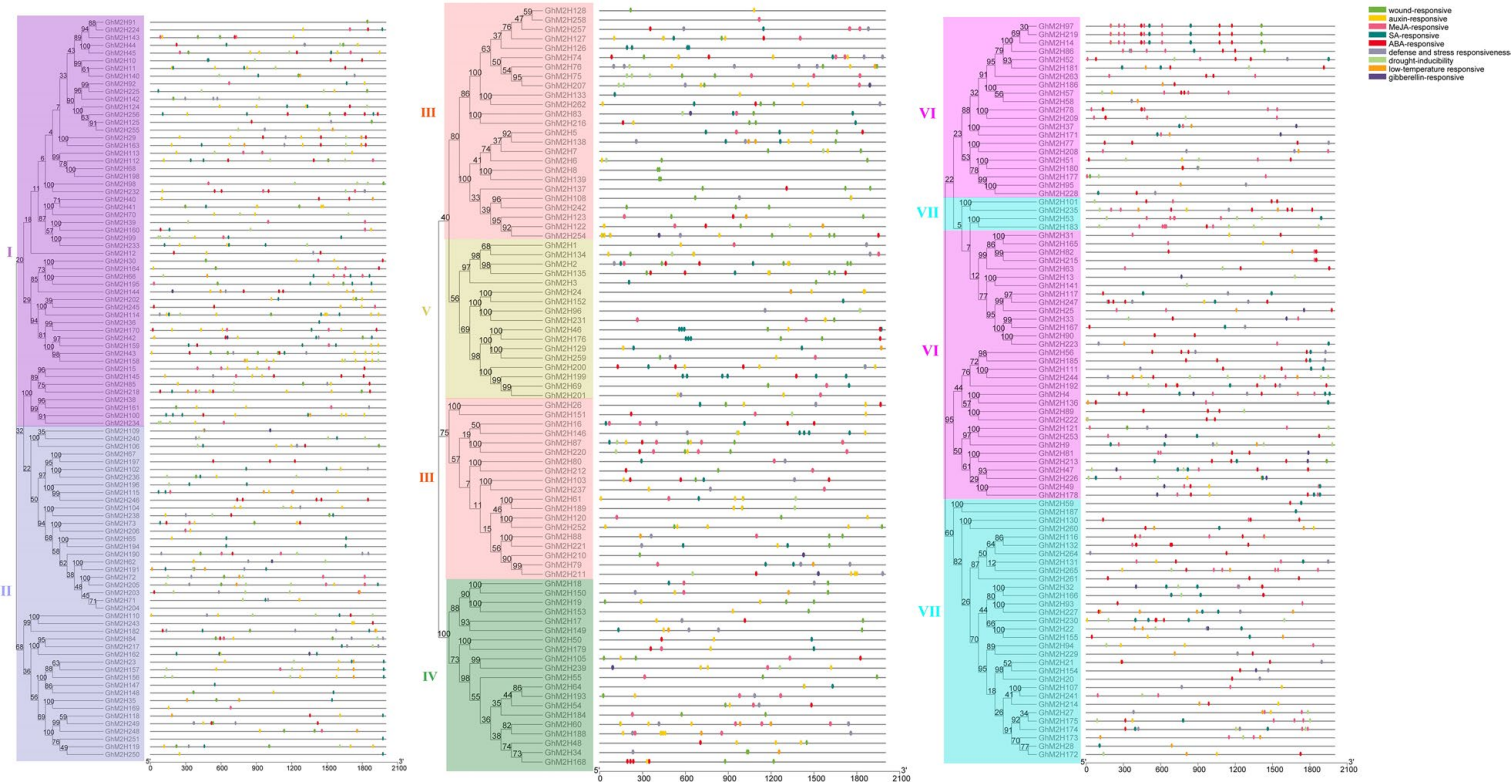
Promoter regions of members of the *Gossypium hirsutum GhM2H* gene family respond to plant hormones and cis-acting elements involved in stress response

Promoters can interact with transcription factors to control the start time and degree of gene expression. The cis-acting element is located in the promoter region of the gene and can be used as a reference for stress response and tissue specificity in different environments [33]. Therefore, analyzing the *GhM2H* promoter region is helpful to explore the potential functions of genes. We used the 2000 bp DNA sequence in the upstream region of *GhM2H* as the promoter, and used the online tool PlantCARE to predict the cis-acting elements (Fig. 5). A large number of cis-acting elements in the promoter region were detected, and selected cis-acting elements related to plant hormones and abiotic stress for further analysis.

For plant hormones, ABA response components, SA response components, gibberellin response components, MeJA response components and auxin response components were selected; and components that respond to abiotic stress include defense and stress responsiveness, wound-responsive components, drought-inducibility components and low-temperature responsive components. More than half of *GhM2H* members contain ABA-responsive elements and MeJA-responsive elements, and only 18 genes are involved in trauma response. We believed that these genes participate in abiotic stress response together with hormone response. Unexpectedly, five *GhM2H* (*GhM2H* 38, *GhM2H* 67, *GhM2H* 68, *GhM2H* 198, *GhM2H*204) did not find the components we need. It is speculated that they may have lost their corresponding functions during the evolution. Through promoter analysis, we can summarize the response mechanism of genes to different plant hormones and abiotic stress.

### Expression profiles of *GhM2H* genes under different abiotic stress

**Fig. 6:**
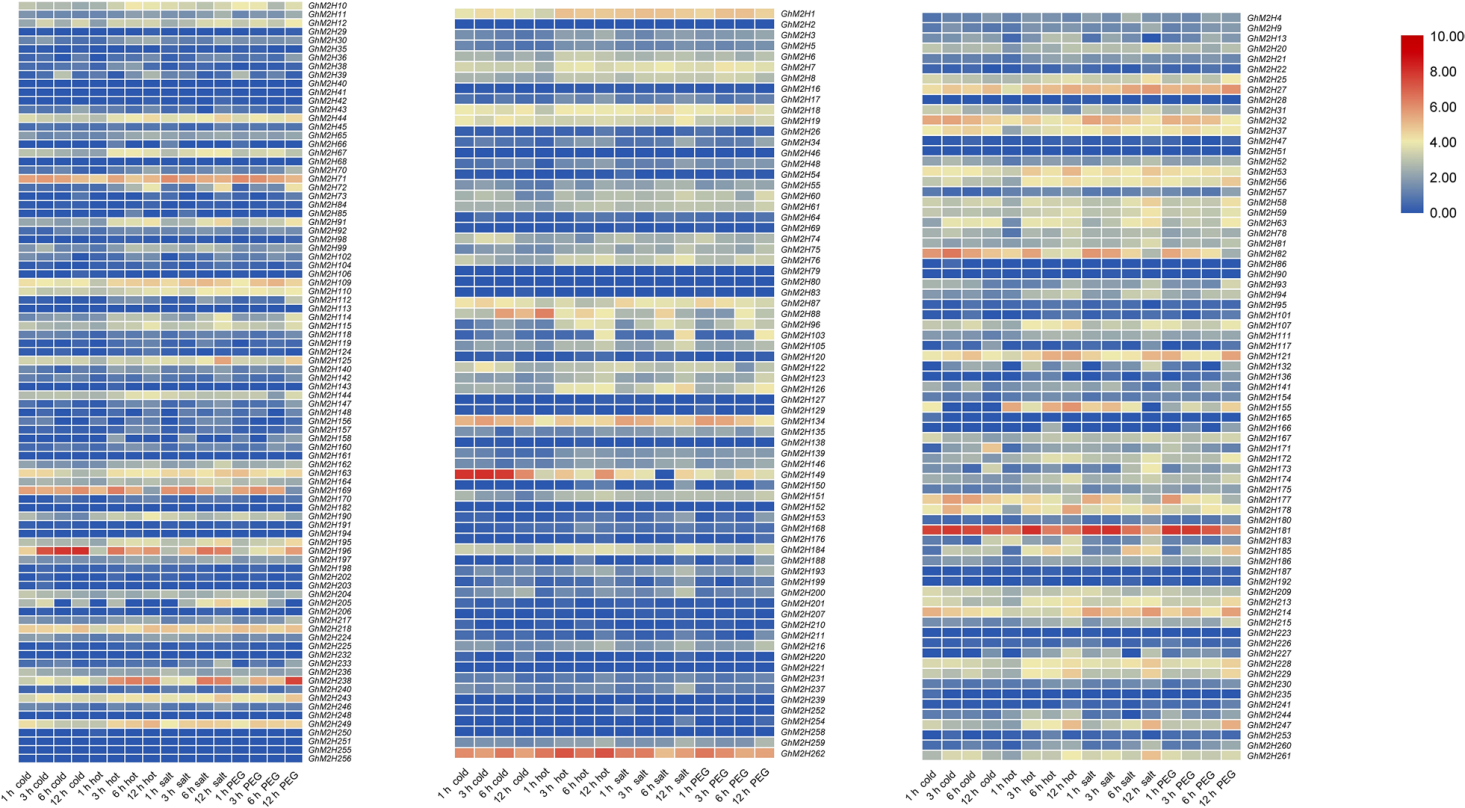
Expression profiles of members of the *GhM2H* gene family in *Gossypium hirsutum* under abiotic stress (cold, heat, salt, PEG)

In order to understand the response mechanism of *GhM2H* to abiotic stress, we downloaded RNA-seq data (PRJNA248163) from the NCBI database to analyze the expression patterns of these genes under various stress (cold, heat, salt and PEG). A total of 231 genes of FPKM were found in the RNA-seq data, and plotted heat maps based on the expression levels of these genes under cold, heat, salt and PEG stress (Fig. 6). The results showed that nearly half of *GhM2H* had no obvious differential expression under a variety of abiotic stress, and some genes were strongly induced under multiple stress and had obvious differential expression, such as *GhM2H*71, *GhM2H*169, *GhM2H*238, *GhM2H*262, *GhM2H*181, etc. We found that gene expression from the same clade is not similar. Interestingly, some genes are only induced by specific stress. For example, *GhM2H*149 has high expression under cold stress, but is not strongly induced by other stress; *GhM2H*155 has almost no expression under cold stress, but occurs under heat stress. The number of *GhM2H* significantly differentially expressed genes under different stress was calculated. There were 101 *GhM2H* genes differentially expressed under cold treatment, 120 *GhM2H* genes differentially expressed under high temperature treatment, 94 and 113 *GhM2H* genes under PEG treatment and salt stress, respectively. Under different stress conditions, gene expression levels changed, indicating that *GhM2H* members were involved in the regulation of abiotic stress.

### qRT-PCR analysis of *GhM2H* genes under different abiotic stress

**Fig. 7:**
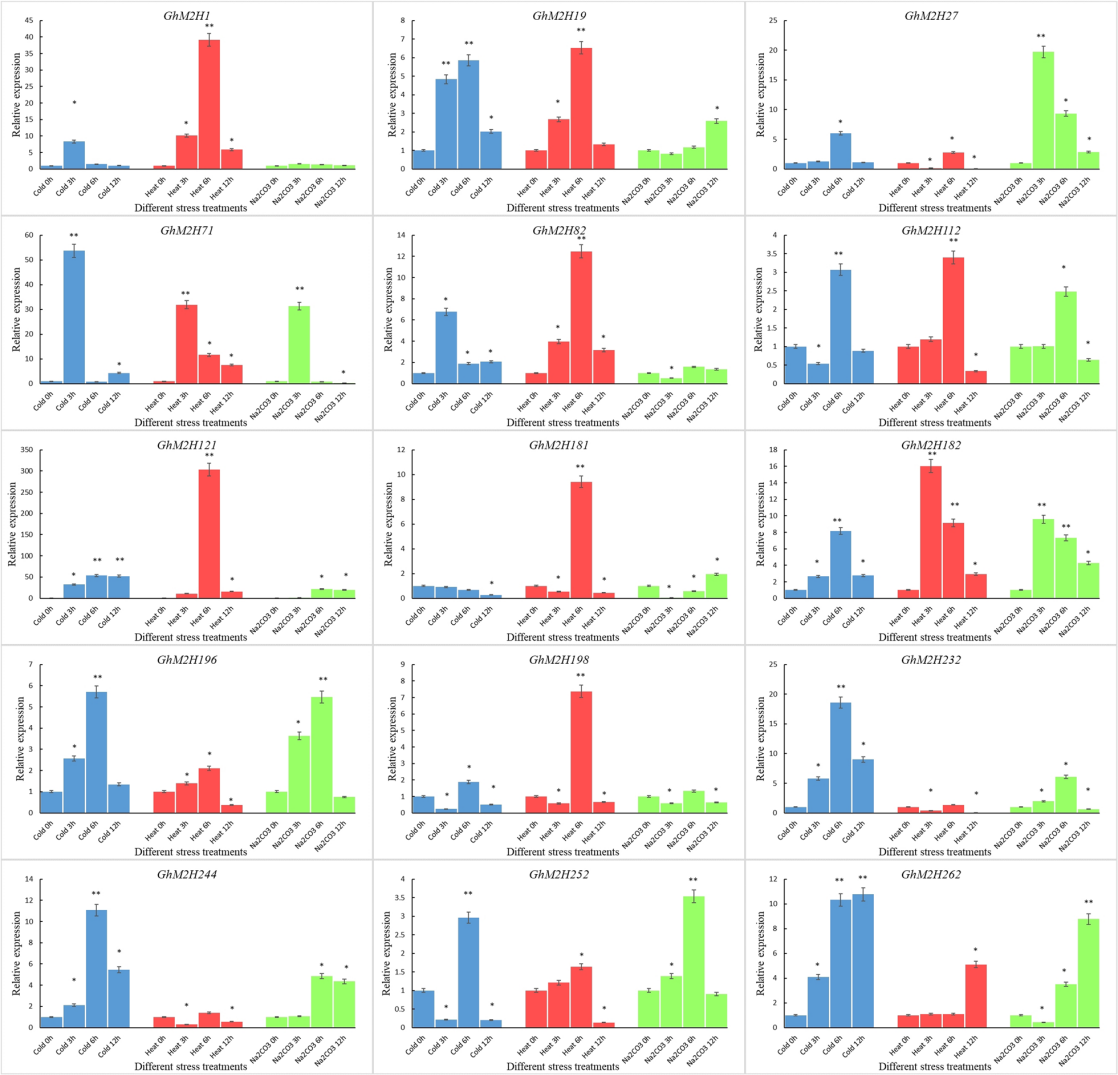
The qRT-PCR analysis of *GhM2H* family members under different stress

As we know, plant melatonin is widely involved in plant responses to abiotic stresses. In rice, AK067086, AK065790, AK119413 and AK101447 encode proteins with melatonin 2-hydroxylase activity. To investigate the role of *GhM2H*s in melatonin involvement in abiotic stress, we performed expression analysis of *GhM2H198*, *GhM2H232*, *GhM2H252*, *GhM2H244*, *GhM2H112*, *GhM2H121*, *GhM2H182*, which are homologous to rice M2H genes, and *GhM2H262*, *GhM2H27*, *GhM2H196*, *GhM2H*2h*181*, *GhM2H82*, *GhM2H71*, *GhM2H1*, *GhM2H19*, which are from the same family in which *GhM2H* is present (Fig. 7). Under the three abiotic stresses, most genes were differentially expressed under stress, but not in response to all stresses. Such as *GhM2H19*, *GhM2H71*, *GhM2H82*, *GhM2H12*, *GhM2H182*, and *GhM2H196* were significantly differentially expressed under all three stresses; *GhM2H1*, *GhM2H121*, *GhM2H181* in response to high-temperature stress; *GhM2H1*, *GhM2H82* were not responsive to Na_2_CO_3_ stress; *GhM2H27*, *GhM2H232* were insensitive to high temperature stress.

### Changes in melatonin content and expression of *GhM2H*s in cotton under salt stress

**Fig. 8:**
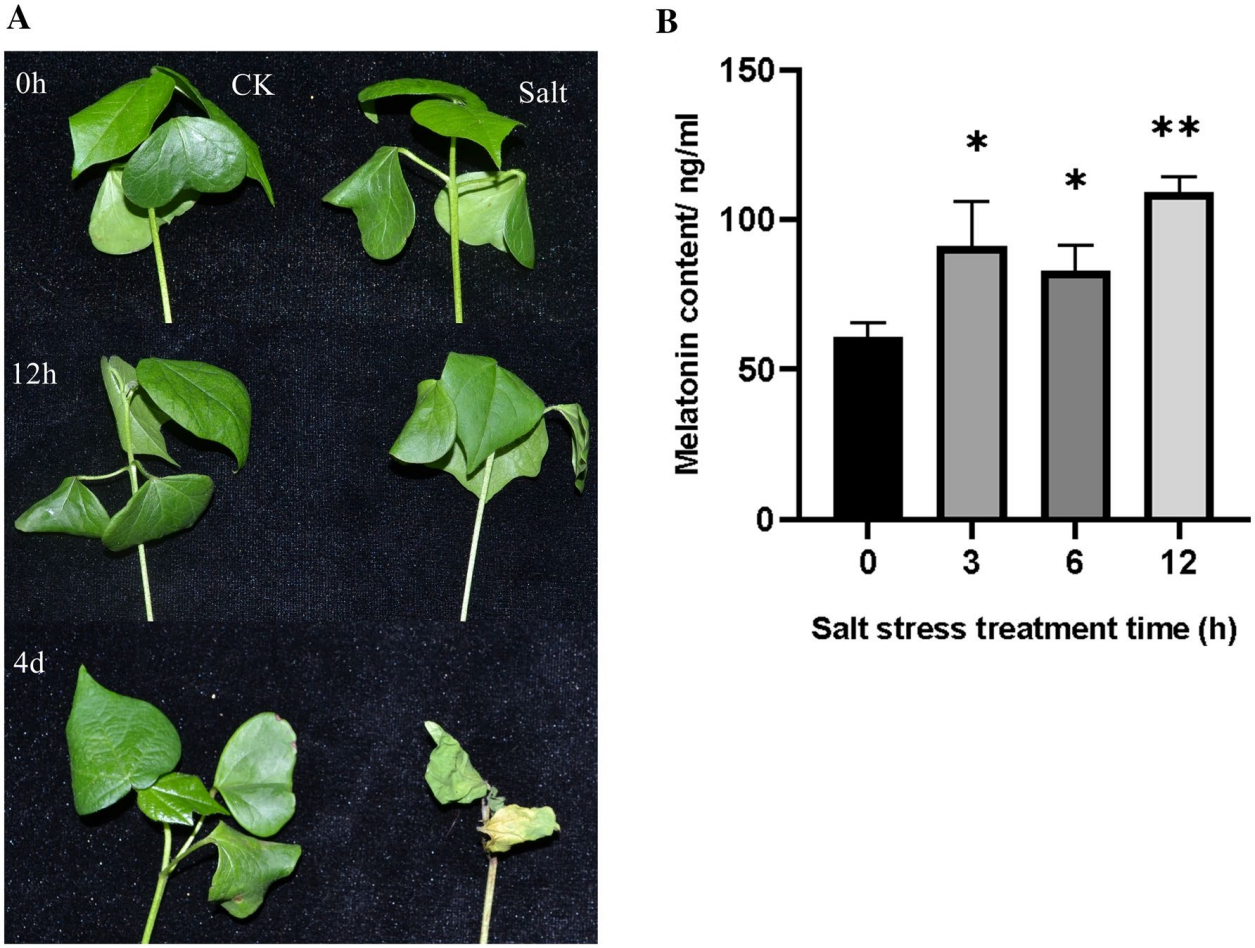

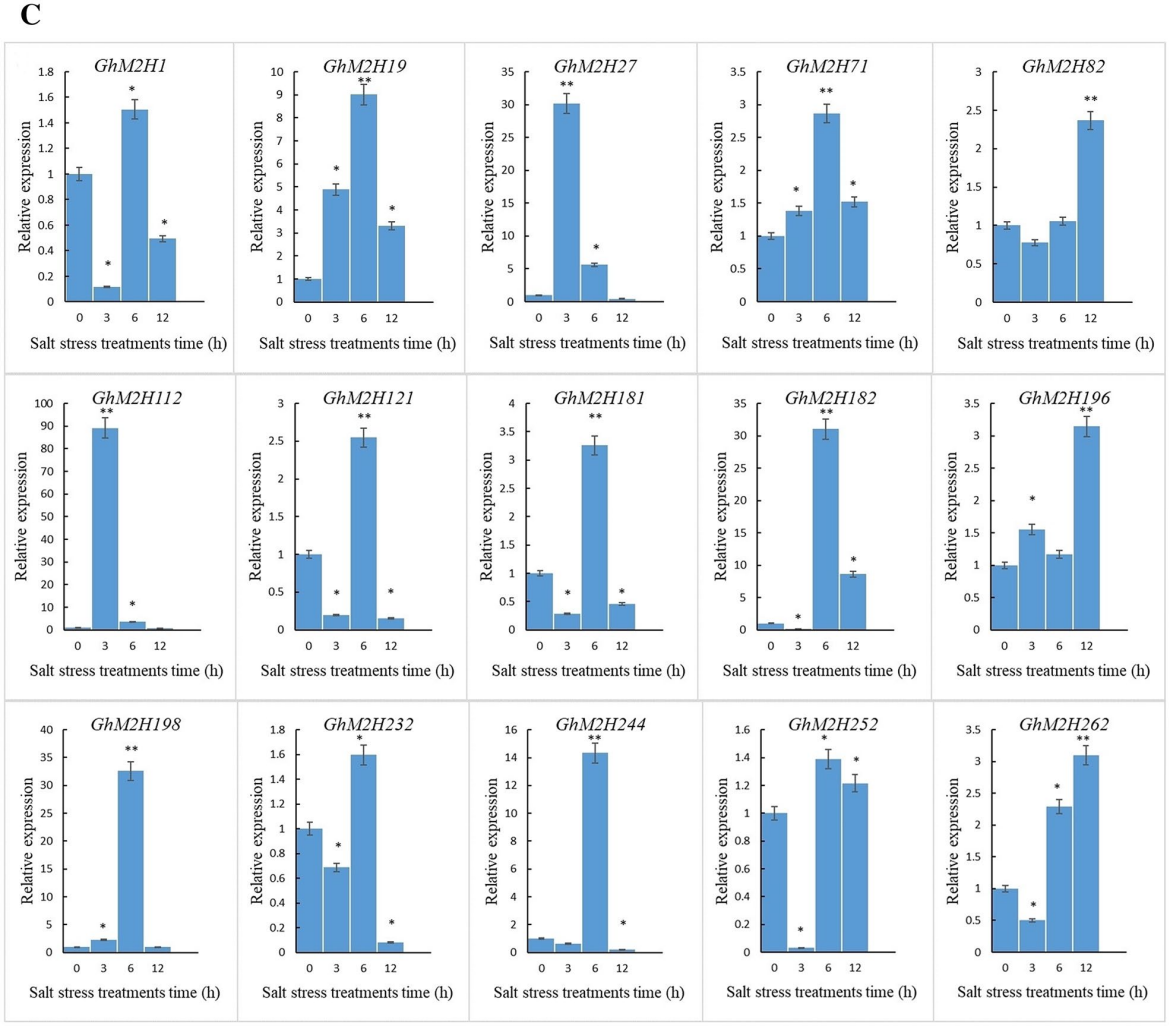
Analysis of melatonin content and *GhM2H*s expression in cotton under Salt Stress.** A** Effects of salt stress on cotton phenotype.** B** Melatonin content in cotton under Salt Stress.** C** Amount of GhM2Hs expressed in cotton under Salt Stress

To explore whether the changes in melatonin content were associated with the changes in the expression of *GhM2H*s under salt stress, we detected the changes in the melatonin content and the expression of *GhM2H*s in cotton under salt stress. Salt stress caused severe effects on the growth and development of cotton seedlings (Fig. 8A). When treated with salt stress for 12 h, the cotyledons of cotton seedlings lost luster and wilted slightly, and the leaves became thinner. With increasing salt stress time, cotton seedlings true leaves appeared wilted. At five days of salt stress treatment, seedling cotyledons were completely detached, true leaves wilted, and seedlings died. The level of melatonin in cotton showed a changing trend of: Rise-Fall-rise (Fig. 8B). Both *GhM2H*s were induced to undergo differential expression under salt treatment, and most genes showed an up-down trend (Fig. 8C), and *GhM2H82*, *GhM2H196*, and *GhM2H262* were up-regulated with 12 h salt treatment. In contrast to the changing trend of melatonin level, the homologs of rice M2H genes, *GhM2H121*, *GhM2H182*, *GhM2H198*, *GhM2H232*, *GhM2H252*, and *GhM2H244*, were all up-regulated at 6 h and down regulated at 12 h of salt stress.

### Effect of exogenous melatonin on *GhM2H*s expression

**Fig. 9:**
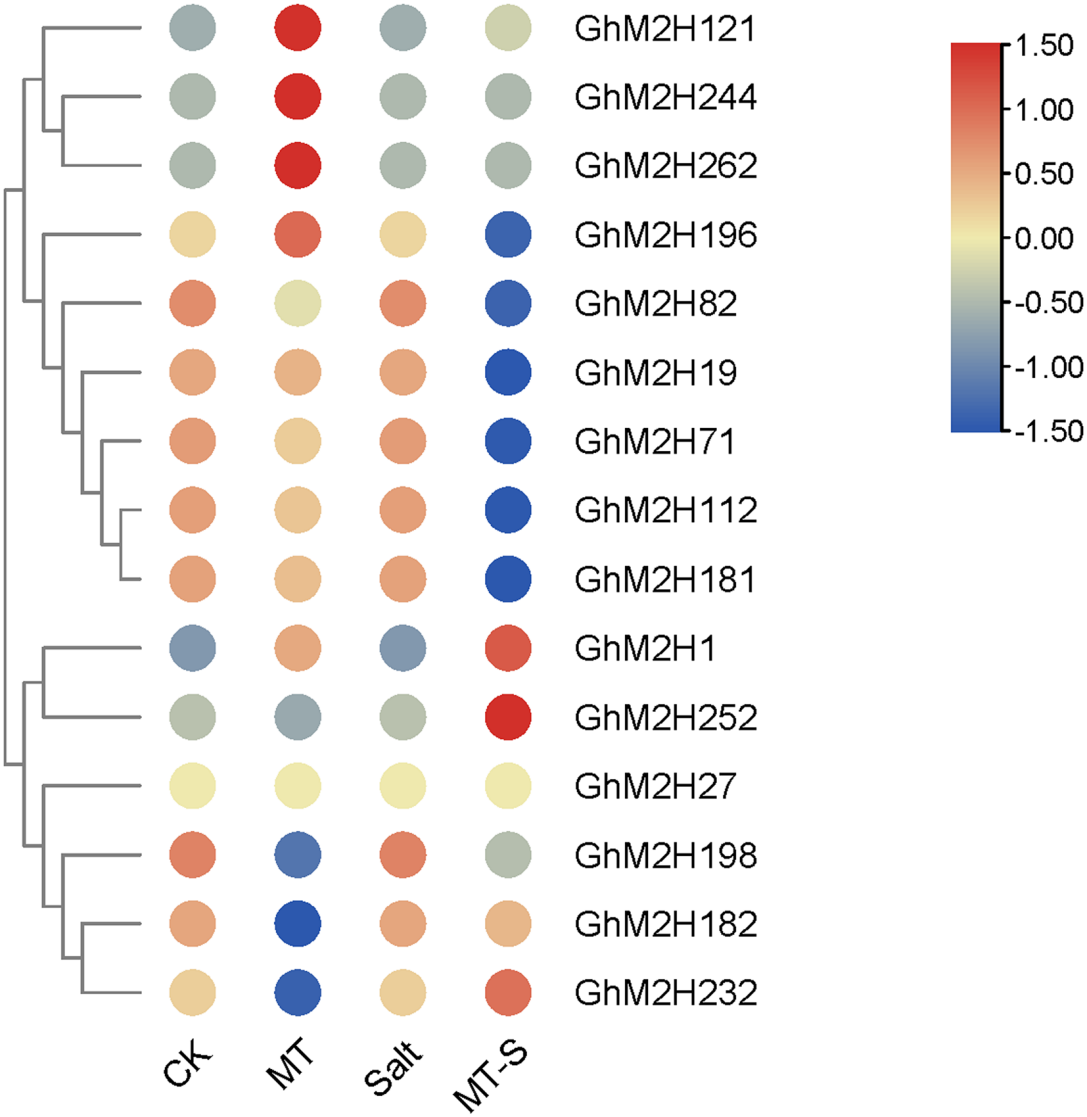
Effect of exogenous melatonin on the expression of *GhM2H*s

Exogenous application of melatonin has been reported to improve cotton salt tolerance [34]. To explore the effects of exogenous melatonin on *GhM2H*s expression, we examined the relative expression amounts of some genes in cotton *GhM2H* family members in response to melatonin under salt stress (Fig. 9). Some *M2H* genes were differentially expressed (*GhM2H121*, *GhM2H244, GhM2H262*, *GhM2H196* were up-regulated, *GhM2H198*, *GhM2H182*, *GhM2H232* were down-regulated) were induced by melatonin, and *GhM2H19*, *GhM2H71*, *GhM2H112*, *GhM2H181*, and *GhM2H27* were not induced by melatonin. Under salt stress, *GhM2H196*, *GhM2H82*, *GhM2H19*, *GhM2H71*, *GhM2H112*, *GhM2H18*, *GhM2H198* down regulated expressions were induced by melatonin; *GhM2H1*, *GhM2H252*, and *GhM2H232* were induced to upregulate expression by melatonin, and *GhM2H27* did not respond to exogenous melatonin.

## Discussion

During the growth and development of cotton, it is often subjected to some abiotic stress, such as drought, salinity, high temperature, and low temperature. As a new type of plant hormone, melatonin has the function of regulating plant growth and stress response. At present, the research on melatonin mainly focuses on its synthesis pathway, and there are few reports on the research on the downstream metabolic pathway of melatonin. This study took *Gossypium hirsutum* as the research object, carried out a whole genome identification of *M2H* genes, and explored the structural characteristics, phylogenetic relationships, evolutionary relationships, expression patterns and responses to various abiotic stress of members of the *M2H* gene family. This study laid the foundation for further exploration of the metabolic pathways of melatonin.

In our study, we identified 265 *M2H* in *Gossypium hirsutum*, the phylogenetic tree (Fig. 1) these genes can be divided into seven clades, clade I for almost all the plants in a *M2H* highest number one branch, the *M2H* number of clade IV is the least. The number of *M2H* in *Gossypium hirsutum* is much higher than that of other plants except *Gossypium barbadense*. This is because *Gossypium hirsutum* has double genome compared with diploid plants, which also indicates that *M2H* is highly conserved in the evolutionary process and has undergone large-scale expansion in higher plants. Motif analysis shows that most *GhM2H* in the same clade seem to have similar motif distribution, which provides further support for their clustering in the evolutionary tree. In our motif analysis results (Fig. 4), most GhM2H proteins contain Motif10, 2, 6, 4, 7, 1, 5, 8. These motif domains represent a part of the M2H domain. Almost all GhM2H proteins of clades I, II, and III consist of 10 motifs arranged in a similar manner, indicating that the protein structure is highly conserved in a specific clade. The composition pattern of motif differs from the grouping of *M2H* in different clades, which may account for the functional specificity of M2H proteins in different categories. In the analysis of gene structure, 74 genes have no introns. These genes with fewer introns can evolve rapidly through replication or reverse transcription, and then integrate into the genome [35]. Genes with fewer introns are commonly found in the genomes of higher eukaryotes [36]. Genes that are closely related have similar gene structures, which may be the result of a series of gene duplications [37]. We predicted the cis-acting elements in the *M2H* promoter region and screened the elements related to plant hormone response and abiotic stress response. Plant hormones may be involved in the regulation of upstream *M2H* genes. More than half of *GhM2H* members contain ABA-responsive elements and MeJA-responsive elements. ABA and MeJA may be important signals regulating the *GhM2H* family. Chromosome mapping results showed that the *M2H* gene distribution in *Gossypium hirsutum* is uneven, which may be caused by tandem duplication or fragment duplication during evolution.

*GhM2H* has repetitive events. For repetitive genes, the aligned sequence must contain at least 70% of the homologous part and cover more than 80% of its total length [38]. Gene replication events in the genome can be divided into two types, namely tandem replication and segmental replication. The distribution of two or more genes on the same chromosome is defined as tandem duplication, and the distribution of these genes on different chromosomes is considered segmental duplication [32]. In the results of collinearity analysis (Fig. 3), we found that 40 pairs of duplicate genes had tandem duplication, and 262 pairs of duplicate genes had fragment duplication in *Gossypium hirsutum*. The occurrence of fragment duplication and tandem duplication promotes the expansion of the *M2H* family. Since the number of fragment duplications is much higher than that of tandem duplication, it indicates that fragment duplication is the main driving force leading to the amplification of *M2H* genes in the evolutionary process [39]. As a tetraploid plant, *Gossypium hirsutum* doubles the size of the *M2H* gene family through fragment and whole genome duplication (WGD) and a small number of tandem duplications. In order to determine the selective pressure acting on gene pairs encoding *GhM2H* homologous proteins at the protein sequence level, we calculated the Ka/Ks values of 233 pairs of genes in *Gossypium hirsutum*. The Ka/Ks values of these genes are all less than 1, indicating that these gene pairs are in progress. Purification selection tends to eliminate harmful mutations during evolution. Under abiotic stress, stress response genes are induced to adapt to various developmental and physiological changes [40]. In the differential expression analysis of *GhM2H*, we found many highly expressed genes, such as *GhM2H*71, *GhM2H*169, *GhM2H*238, *GhM2H*262, *GhM2H*181, etc., indicating that their expression is controlled by abiotic stress. Most of these highly expressed genes contain ABA-responsive elements and MeJA-responsive elements. These cis-acting elements are present in the promoters of highly expressed genes, indicating that they may be involved in the stress response. We also found that most of the repetitive gene pairs show similar expression trends under abiotic stress. Duplicate genes play an important role in adapting to the external environment during evolution and maintaining the stability of the genetic system when attacked by environmental stimuli.

Under abiotic stress, M2H gene family members are induced to express, but not in response to all stresses. These results illustrated that the family members in which *GhM2H* is located are involved in regulating abiotic stress, and part of the genes respond to specific stresses. Given the opposite trend of melatonin level changes and expression changes of *GhM2H*s under salt stress, we speculated that *GhM2H*s regulated cotton melatonin levels by breaking down melatonin. Since the endogenous melatonin level rises in cotton seedlings in response to stress, in order for plants to regulate the endogenous melatonin level, the expression of genes with M2H enzymatic activity is upregulated to accelerate endogenous melatonin breakdown; whereas plants suppress endogenous melatonin breakdown by decreasing M2H related expression to increase the level of endogenous melatonin as the stress time increases, these M2Hs are highly likely to be candidate genes for the breakdown of endogenous melatonin into 2-Ω in cotton. As the homologous gene of rice AK119413, *GhM2H*198 also showed the tendency of first rising and then falling under the stress of Heat, Cold, Na_2_CO_3_, and *GhM2H198* might be one of the major genes to decompose melatonin in cotton. Based on these data, we speculated that these genes may have a very complex regulatory network to regulate endogenous melatonin level.

## Conclusions

For the first time, we comprehensively identified *M2H* in the *Gossypium hirsutum* genome. A total of 265 *GhM2H* genes were identified. With the support of phylogenetic tree, gene structure and motif composition, *M2H* proteins were divided into 7 clades. Collinearity analysis indicates that this gene family has undergone tandem replication, fragment replication, and genome-wide replication during evolution. Through the analysis of cis-acting elements in the promoter region, we speculate that some genes are regulated by ABA and MeJA and participate in abiotic stress response. Through expression analysis, the differential expression pattern of *GhM2H* under abiotic stress was revealed, indicating that they play a role in stress response, partial genes respond to specific stresses. M2H showed opposite change trend with melatonin under salt stress, *GhM2H*s might regulate endogenous melatonin level through a very complex regulatory mechanism, and some genes in the *GhM2H* gene family were regulated by exogenous melatonin. The results of this study provide a reference for further analysis of the function of *GhM2H* gene and exploration of melatonin metabolism.

## Materials and methods

### Identification of *GhM2H *Gene Family Members in *Gossypium hirsutum*

In order to identify the members of the *GhM2H* gene family, we download the protein sequence and genome data of *Gossypium hirsutum* from CottonFGD (https://cottonfgd.org) [41], The nucleotide sequence and protein sequence of *Oryza sativa M2H* (AK067086, AK065790, AK119413, AK101447) was used as the query sequence for comparison in the local database [18]. The *Gossypium hirsutum* genes with e-value values less than 1e-5 were screened for further analysis. The conserved domains of *M2H* protein 2OG-FeII_Oxy and DIOX_N (PF03171.20, PF14226.6) were used for further analysis using the Pfam database (https://pfam.xfam.org/) and the online Tools CD-Search Tool (https://www.ncbi.nlm.nih.gov/Structure/bwrpsb/bwrpsb.cgi), genes with incomplete domains were manually eliminated. According to the position of the gene on the chromosome, we renamed the gene *GhM2H*1-*GhM2H*265. The online tool ExPASy-ProtParam was used to analyze the physical and chemical properties of the *GhM2H* gene (https://web.expasy.org/protparam/) [42]. To understand the subcellular localization of *M2H* protein, we used online sites to make predictions, for example, TargetP (http://www.cbs.dtu.dk/services/TargetP/) [43], WOLF-PSORT (https://wolfpsort.hgc.jp/) and CELLO *ver*. 2. 5 (http://cello.life.nctu.edu.tw/) [44].

### Phylogenetic analysis

To study the evolutionary relationship between *M2H* genes in different species, we used the above method to obtain the homologous genes of *M2H* in the other three cotton species (*Gossypium barbadense, Gossypium arboreum, Gossypium raimondii*). The conserved domains 2OG-FeII_Oxy and DIOX_N (PF03171.20, PF14226.6) were used as keywords, online database Phytozome v12. 1 was used to compare homologous genes of other species (*Theobroma cacao*, *Arabidopsis*, *Oryza sativa*) (https://phytozome.jgi.doe.gov/pz/portal.html), the protein sequences of the four cotton species and *M2H* family members of *Theobroma cacao*, *Oryza sativa* and *Arabidopsis* were inputted to MEGA7.0. MEGA7.0 was used to construct rootless phylogenetic trees with the following parameters: MEGA7.0 was used to construct the interspecific phylogenetic tree of M2H protein and the intra-specific phylogenetic tree of GhM2H in upland cotton, and the parameters are as follows: Bootstrap replication:1000, model/method: P-disrance, and all /Missing Data Treatment: Partial deletion. [45].

### Chromosomal location

W**e** download the whole genome annotation file of *Gossypium hirsutum* in CottonFGD (https://cottonfgd.org/about/download/annotation/gene.Ghir.HAU.gff3.gz), used the software TBtools to visualize the chromosome positions of *GhM2H* members [46].

### Collinearity analysis of *M2H*s in four cotton species

The MCScanX software was used to analyze the collinearity of the four cotton species duplication gene pairs of *Gossypium hirsutum*, *Gossypium barbadense*, *Gossypium arboreum*, *Gossypium raimondii* [47]. The collinear and homologous chromosomal regions among four cotton species were visualized using advance Circos package in TBtools[46].

### Calculation of selection pressure

To determine the selection pressure, the software TBtools was used to calculate the Ka (non-synonymous substitution) and Ks (synonymous substitution) rates of repeated genes [48].

### Analysis of motif and gene structure of conserved proteins

We used genome-wide annotation files of *Gossypium hirsutum* downloaded from cotton database CottonFGD to obtain the genetic structure of *GhM2H* members, we used online software MEME to predict the motif of genes (http://meme-suite.org/tools/meme), parameters are as follows: the maximum number of motifs in each gene is 10, and the remaining parameters are set by default. TBtools was used to draw the evolutionary relationship, gene structure and motif composition association analysis of *GhM2H* [48].

### Analysis of *GhM2H* promoter regions

The 2000 bp DNA sequence of the upstream region of *GhM2H* from the CottonFGD database was obtained as a promoter [49]. We used the PlantCARE database to predict the cis-regulatory elements in the promoter region of the *GhM2H* gene (http://bioinformatics.psb.ugent.be/webtools/plantcare/html/), selected cis-acting elements related to plant hormones and abiotic stress for further analysis, and use the software TBtools to visualize the results [48].

### Analysis of differentially expressed genes

In order to study the expression patterns of the *GhM2H* gene family, we downloaded RNA-seq data (PRJNA248163) from the NCBI database to analyze the expression levels of these genes under cold, heat, salt and PEG stress [50], used the software TBtools to visualize the expression patterns of *GhM2H* under different abiotic stress [48].

### Stress treatment and qRT-PCR analysis

The seeds of Zhong 9807 were sown on a 1:1.5 medium substrate of sand and vermiculite, and grown in an indoor incubator at 25 °C for 16 h during the day and 8 h at night. In order to study the expression patterns of *GhM2H* gene under different stress, cotton seedlings were treated under different stress at the three-leaf stage, and the plants were treated at 4℃, 40℃, Na_2_CO_3_ (50 mM) and NaCl (200 mM), respectively. Leaves were collected at 0, 3, 6 and 12 h respectively for RNA extraction, three replicates, and treated with water as control. Total RNA was extracted with EASYspin Plus Plant RNA rapid separation kit (Aidlab Co., LTD., Beijing, China). The pure RNA was reverse-transcribed using TransScript® II one-step gDNA removal and cDNA synthesis supermix (TransGen Biotech Co., LTD, Beijing, China) according to the manufacturer's instructions. GhM2Hs and some family members highly homologous to M2H in rice were selected for expression analysis. The GenScript online tool (https://www.genscript.com/tools/real-time-pcr-taqman-primer-design-tool) was used to design qPCR-specific primers. All primer sequences are shown in Additional file 3. qRT-PCR assays were performed on the Bio-Rad 7500 fast fluorescence quantitative PCR platform with TransStart® top green qPCR supermix (TransGene Biotech Co., LTD, Beijing, China) in accordance with the manufacturer's protocol. In three biological replicates, the 2-ΔΔCt method was used to measure the relative expression level of the GHM2H genes.

### Determination of melatonin content in cotton

In order to explore the change of melatonin content under salt stress, cotton was treated with 200 mM NaCl and sampled at 0,3,6,12 h to detect the melatonin content. We took the samples needed to determine the endogenous melatonin content, and used the Plant Melatonin (MT) ELISA Kit (Ziker, ZK-P7490, Shenzhen, China) to measure the endogenous melatonin content. The assay was performed according to the instructions of the Plant Melatonin (MT) ELISA Kit, with three biological replicates for each sample.

### Treatment of cotton with exogenous melatonin and salt stress

In order to explore the effect of increasing melatonin level on the expression of GhM2Hs, 20 µM melatonin was used to treat three leaf cotton seedlings. The leaves were sprayed once a day for three consecutive days. They were grown in an indoor incubator at 25 ℃ for 16 h in the day / 8 h at night [34]. Melatonin treatment at 0 µM concentration was used as control. Melatonin treated cotton seedlings and control seedlings were treated with 100 mM NaCl solution for 12 h. Samples were taken respectively for three biological replicates. Total RNA was extracted by EasySpin plus plant RNA rapid separation Kit. The selected 15 GhM2Hs were examined for expression.

## Supplementary Information

**Additional file 1**: *Gossypium hirsutum* 265 GhM2H basic information.

**Additional file 2**: Calculation of non-synonymous (Ka) to synonymous (Ks) substitution.

**Additional file 3**: Primer information for real time fluorescence quantification.

## Data Availability

Not applicable.

## Abbreviations

M2H: Melatonin 2-hydroxylase
ABA: Abscisic acid
MeJA: Methyl jasmonate
ASMT: *N-*Acetylserotonin methyltransferase
2-ODD family: 2-Oxoglutarate-dependent dioxygenase
Ga: *Gossypium arboretum*
Gb: *Gossypium barbadense*
Gh: *Gossypium hirsutum*
Gr: *Gossypium raimondii*
