# Supplementary material for: Genome-wide identification and characteristic analysis of the downstream melatonin metabolism gene GhM2H in Gossypium hirsutum L
Source: Biol Res. 2021 Nov 4;54:36. doi: 10.1186/s40659-021-00358-y (PMC8567562; doi:10.1186/s40659-021-00358-y)
Supplement: Supplementary file 3 — Additional file 3: Primer information for real time fluorescence quantification. [file 40659_2021_358_MOESM3_ESM.docx]

| Gene | Forward Primer 5’-3’ | Reverse Primer 5’-3’ |
| --- | --- | --- |
| *GhM2H1* | ACTATCCTCCATGCCCTTTCCC | CCGTTCCGAGTTCACCACCA |
| *GhM2H19* | GCAATGGCCGCTACAAGAGTG | TGTGGTGCAGGAGCAATGGT |
| *GhM2H27* | GCTCCAACACTTGCTGATCC | GGAGGGCTTTAAGACCCAGT |
| *GhM2H71* | CCTTCCAACCCTCCTTCCTTCA | TGTCCAGGCAATCCGTATGTCA |
| *GhM2H82* | AGTGTGATGCACCGTGTGATTG | ACCTTGGCTCCTTGGCTTGG |
| *GhM2H112* | TGGTCTAGCACCACACTCAG | AGGGATGGGTTTAACAGGCA |
| *GhM2H121* | CAGGCAGCTACATTGTTCCC | TGATGATCTGCTGCCTGGAA |
| *GhM2H181* | CTTCGCCAACTCCAAGACCTCT | TTCCTTCTCCGCCTCGTTATGG |
| *GhM2H182* | AGTGTGATGCACCGTGTGATTG | ACCTTGGCTCCTTGGCTTGG |
| *GhM2H196* | CTGTTGCTTCCTTCCTCTGC | CCCTGTATACGGCTCCACAT |
| *GhM2H198* | CGTCTCATGAGGCTGATTGC | TGGAATCAGAGTGTGGTGCT |
| *GhM2H232* | TGTTACGTCGTTTCGCCTTC | CTATGGCGACAAGCTTCACC |
| *GhM2H244* | ATGGGAAACTACGCAAAGGC | GTGAGGTCTGGTTCAGGACA |
| *GhM2H252* | GTGTTCTCCATCGAGCTGTG | GCCAATGGGTTCTGACCATC |
| *GhM2H262* | AGGTATGCCACCACACTCAGAT | CTATGGACAGCCGCTTGTTCTC |

**Attachment 3** Primer information for real time fluorescence quantification
